# Structural Analysis of Regenerated Cellulose Textile Covered with Cellulose Nano Fibers

**DOI:** 10.3390/polym17152015

**Published:** 2025-07-23

**Authors:** Ayaka Yamaji, Yui Okuda, Chikaho Kobayashi, Rikako Kurahashi, Kyoko Kazuma, Kazuki Chiba, Mitsuhiro Hirata, Yuka Ikemoto, Keiichi Osaka, Jiacheng Gao, Harumi Sato, Go Matsuba

**Affiliations:** 1 Graduate School of Organic Material Science, Yamagata University, 4-3-16 Jonan, Yonezawa 992-8510, Yamagata, Japan; mountain.road.0907@gmail.com (A.Y.); yui0108@yz.yamagata-u.ac.jp (Y.O.); tyr78369@st.yamagata-u.ac.jp (C.K.); tmr51135@st.yamagata-u.ac.jp (R.K.); 2 Yamagata Research Institute of Technology, 2-2-21 Shoei, Yamagata City 990-2473, Yamagata, Japan; kazumak@pref.yamagata.jp (K.K.); chibaka@pref.yamagata.jp (K.C.); hiratam@pref.yamagata.jp (M.H.); 3 JASRI/SPring-8, 1-1-1 Koto, Sayo-cho 679-5198, Hyogo, Japan; ikemoto@spring8.or.jp (Y.I.);; 4 Graduate School of Human Development and Environment, Kobe University, 3-11 Tsurukabuto, Nada-ku, Kobe 657-8501, Hyogo, Japan; 219d406d@stu.kobe-u.ac.jp (J.G.); hsato@tiger.kobe-u.ac.jp (H.S.)

**Keywords:** regenerated cellulose, cellulose nanofiber, X-ray scattering, FT-IR

## Abstract

Cellulose nanofiber (CNF) treatments can enhance the structure and performance of regenerated cellulose fibers. This study investigates the effects of CNF treatment on the mechanical properties, water absorption behavior, and humidity dependence of regenerated cellulose fibers. Tensile testing demonstrated that CNF-treated fibers exhibit improved elasticity and reduced swelling in aqueous environments. Scanning electron microscopy revealed the adsorption of CNF components onto the fiber surfaces. Microbeam X-ray diffraction indicated structural differences between untreated and CNF-treated fibers, with the latter containing cellulose I crystals. Small-angle X-ray scattering revealed alterations in the internal fibrillar structure due to CNF treatment. FT-IR spectroscopy highlighted humidity-dependent variations in molecular vibrations, with peak intensities increasing under higher humidity conditions. Additionally, CNF treatment inhibited water absorption in high-humidity conditions, contributing to reduced expansion rates and increased elastic modulus during water absorption. Overall, CNF treatment enhanced both the mechanical strength and water resistance of regenerated cellulose fibers, making them suitable for advanced textile applications. This study provides valuable insights into the role of CNF-treated fibers in improving the durability and functional performance of regenerated cellulose-based textile.

## 1. Introduction

The textile and garment sector accounts for an estimated 6–8% of total global carbon emissions, approximately 1.7 billion tons per year [1]. A significant portion of this impact stems from synthetic fiber materials, including polyester, acrylic, and nylon, which are derived from petroleum and represent roughly 70% of the global fiber production [2]. As a result, the fiber and apparel industries bear substantial responsibility for advancing a more sustainable society. Despite recognizing these issues, industry practices continue to prioritize the production and use of synthetic fibers to maximize profitability. Nevertheless, ongoing research and development efforts are focused on identifying alternative materials that can mitigate the sector’s contribution to carbon emissions.

Biodegradable plastics, such as polylactic acid, have emerged as promising alternatives for reducing petroleum dependence and environmental impact. Accordingly, numerous studies have examined their fundamental physical properties, improved their molding processability, and enhanced their suitability for fiber production [3,4]. Natural fibers, such as cotton, are also valuable alternatives that contribute toward reducing the reliance on petroleum and environmental burden. However, cotton cultivation requires large amounts of water and pesticides, which can diminish their overall sustainability. Recently, regenerated cellulose fibers—chemically processed natural fibers—have gained attention as promising textile materials due to their enhanced sustainability and high wearing comfort.

Currently, regenerated cellulose fibers account for approximately 6–7% of the global textile market [5,6,7], and their usage is increasing rapidly. For example, some reports discussed special textiles for biomedical applications [8] and additive manufacturing [9]. However, they have inherent limitations. The crystalline packing of the chains in regenerated cellulose fibers is characterized by the anti-parallel chain arrangement of cellulose II, which results in lower mechanical properties when wet compared to the cellulose I structure found in natural fibers. Wet friction can cause regenerated cellulose fibers to break, fluff, entangle, shrink, and delaminate, ultimately leading to fabric damage and discoloration.

Regenerated cellulose has been extensively treated with a shrink-resistant process using urea and formaldehyde. On the other hand, one of the applications of regenerated cellulose fibers is for clothing. Since clothing comes into direct contact with the human body, its chemical safety is of the utmost importance. However, processing using formaldehyde is difficult to accept in the market due to the high concentration of formaldehyde remaining in the fibers. Therefore, the most commonly used method for improving the properties of regenerated cellulose fiber for clothes is processing with glyoxal resin. This resin reacts with the OH groups on the fiber surface, forming cross-links to impart strong shrink-resistant properties. However, when shrink resistance is enhanced, the remained metal catalysis for cross-linking solidifies has some effects of acidic degradation of cellulose molecules, resulting in a trade-off relationship [10,11]. To improve the properties of regenerated cellulose fibers, some researchers have examined the ionic liquid system [12,13,14]. The use of ionic liquids is very costly, and considering their safety for humans, it is extremely difficult to produce regenerated cellulose fibers industrially. In particular, Edgar and Zhang showed research progress in the antibacterial modification of regenerated cellulose fibers [15].

Recently, Tohoku Seiren Co., Ltd. developed a processing method using cellulose nanofibers (CNFs) to improve the functionality of regenerated cellulose fibers [16]. As shown in Figure S1, this CNF treatment alters the material’s response to washing. The treatment enhances the structural integrity of regenerated cellulose fibers by altering their physical properties and characteristics. Furthermore, this CNF treatment technique is very simple and is possible for small manufacturing companies due to improvements in existing dyeing processes. However, the structural origin of these changes remains poorly understood. This study evaluates the correlation between processed regenerated cellulose fibers and their interaction with water by examining elastic modulus, microscopy, microbeam X-ray scattering, and microbeam FT-IR spectroscopy. Our findings show that CNF processing inhibits the water absorption of regenerated cellulose fibers, thereby helping to maintain their mechanical strength under wet conditions.

We hypothesize that cellulose nanofibers are strongly oriented along the surface of regenerated cellulose fibers through hydrogen bonding, forming a coating that enhances water resistance and durability. However, previous studies have shown that when carbon nanotubes are deposited on carbon fibers, they exhibit a completely random orientation and are not necessarily aligned along the fiber axis [17]. This suggests that nanoscale materials do not inherently align with the underlying fiber structure. If our hypothesis can be verified, it would demonstrate that the orientation of CNFs is driven by interactions between the nanofibers and the crystalline regions within the macroscopic fibers. This, in turn, would help elucidate the mechanism by which nanoscale structural control contributes to improvements in the macroscopic properties of regenerated cellulose fibers.

## 2. Materials and Methods

### 2.1. Materials

Regenerated cellulose fibers (Cupro, B4615, Asahi Kasei Corp., Tokyo, Japan) were supplied by Tohoku Seiren Co., Ltd. (Yonezawa, Japan). TEMPO-oxidized CNFs were prepared using a Cellenpia TC-01A system (Nippon Paper Industries Co., Ltd., Chiyoda-ku, Tokyo, Japan) from a 1 wt.% aqueous dispersion. The CNF dispersion was concentrated to 10 wt.%. The CNFs had an average diameter of approximately 3 nm, a length of ~1 μm, and a crystallinity of 70%. The charge density was 1.5 mmol/g, with Na^+^ as the counterion. For CNF treatment, regenerated cellulose fibers were processed using a Beaker dyeing system (LABOMAT, Mathis, Oberhasli, Switzerland) and a sample dyeing padder machine (Daiei Kagaku Seiki MFG Co., Ltd., Kyoto, Japan). The CNF treatment procedure has been described in detail by [16].

### 2.2. Measurements

Tensile tests were conducted using a FAVIGRAPH system (Textechno Herbert Stein GmbH & Co. KG, Mönchengladbach, Germany) at 23 °C. The FAVIGRAPH integrates linear density measurement and tensile testing in a single instrument. The linear density measurement head, based on FAVIMAT+ technology, was positioned adjacent to the tensile testing section [18]. Fibers were manually loaded with appropriate pretension. Tensile testing was performed either 30 or 5 times under an initial load of 100 mg, a gauge length of 10 mm, and a tensile speed of 10 mm/min. Fineness was measured using the resonance method. The number of fibers was 30 samples, and 95% confidence intervals were calculated for each parameter. The washing test was conducted according to the standard washing test (JIS L 1930 C4N) [19] using laundry detergent (JAFET standard detergent, Japan Textile Evaluation Technology Council, Tokyo, Japan). After washing, the test sample was drying in the sun.

Laser optical microscopy was conducted using an LEXT OLS4000 microscope (Olympus Corporation, Hachioji, Tokyo, Japan). Electron microscopic images were obtained using a field-emission scanning electron microscope (FE-SEM) from JEOL Ltd. (Akishima, Tokyo, Japan), located at the Yamagata Research Institute of Technology. Prior to imaging, samples were coated with a 20 nm osmium layer. Secondary electron imaging and elemental analysis were performed at an accelerating voltage of 1 kV in gentle beam mode. All microscopy measurements were carried out at 23 °C.

Microbeam wide-angle X-ray scattering (WAXS) measurements were performed using the BL40XU beamline at SPring-8 (JASRI, Hyogo, Japan) with an X-ray wavelength of 0.10 nm [20]. The sample-to-detector distance was 52.3 mm, with an exposure time of 10 s, and a beam size of 500 nm × 500 nm. An EIGER 1M (Dectris AG, Baden, Switzerland) detector was used for data acquisition. Samples were prepared by isolating individual filaments from the fiber bundle. Each filament was mounted on a metal holder, with the ends secured using tape to prevent bending during measurement. The scattering vector q range was 7.0 to 18 nm^−1^, and its magnitude (*q*) is defined as:

(1) q=4πsinθ/λ

where 2θ is the scattering angle and λ is the X-ray wavelength.

Ultrasmall-angle X-ray scattering (USAXS) and small-angle X-ray scattering (SAXS) measurements were performed at the BL19B2 beamline at SPring-8 [21], with an incident X-ray wavelength of 0.068 nm at 25 °C. The camera lengths for the USAXS and SAXS experiments were set to 40.88 and 3.04 m, respectively. Two-dimensional (2D) USAXS and SAXS profiles were obtained using a PILATUS-2M 2D detector (Dectris AG). The scattering vector *q* ranged from 5 × 10^−3^ to 2.8 nm^−1^. Data processing, including contrast control of the 2D patterns and extraction of 1D profiles from the 2D patterns, was performed using FIT2D software (version 12.077, Andy Hammersley, European Synchrotron Radiation Facility (ESRF), Grenoble, France).

Microbeam FT-IR spectroscopy was performed at the BL43IR beamline at SPring-8 in Hyogo, Japan [22,23]. A Bruker VERTEX 70 spectrometer, coupled with a Hyperion 2000 FT-IR microscope (Bruker Corp., Billerica, MA, USA), was used at 25 °C. An in-house humidity-controlled cell, connected to a humidity generator (HUM-1; Rigaku Corporation, Akishima, Tokyo, Japan), was installed on the IR microscope. A 1-mm-thick barium fluoride window served as the IR window. Detailed descriptions of the microbeam FT-IR spectroscopy can be found in our previous studies [24,25]. Relative humidity (RH) was maintained at ±1% by mixing appropriate proportions of dry and water-saturated nitrogen gas streams using electronic mass flow controllers. Humidity-dependent measurements were conducted after equilibrating the samples to stand for at least two hours. A computer-controlled stepping motor was used to precisely position the sample and ensure reproducibility.

Raman spectra were recorded over the range of 400–100 cm^−1^ using a LabRAM HR Evolution Raman microscope (HORIBA Ltd., Kyoto, Japan) equipped with a Syncerity CCD detector and a 532 nm excitation laser. Measurements were carried out at 23 °C. The spectral resolution, laser power, exposure time, and number of scans were set to 0.5 cm^−1^, 100 mW, 15 s, and 5, respectively [26].

## 3. Results and Discussion

### 3.1. Mechanical Properties

**Figure 1:**
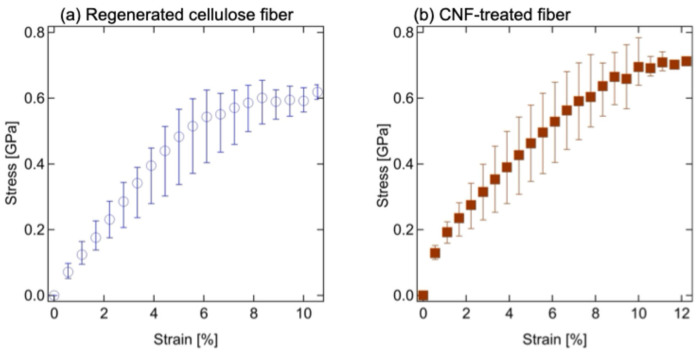
Stress–strain curves of a single filament in (**a**) regenerated cellulose fiber and (**b**) CNF-treated fiber.

The degree of fiber swelling was evaluated. For regenerated cellulose fibers, the diameter increased from 8.47 μm in the dry state to 14.57 μm after swelling, corresponding to a swelling ratio of 1.72. In contrast, CNF-treated fibers had a dry diameter of 9.86 μm and swelled to 16.4 μm, resulting in a swelling ratio of 1.65. These results indicate that CNF treatment reduces water absorption. Additionally, improvements in strength retention and dye fastness were observed. Furthermore, stress–strain curves for individual fibers were also obtained, as shown in Figure 1.

The regenerated cellulose fibers approached the yield point, whereas the CNF-treated fibers exhibited a nearly linear stress–strain curve. The breaking strength of the untreated fibers was 0.319 (±0.011) GPa, and that of the CNF treatment fiber was 0.289 (±0.012) GPa, with a 95% confidence interval as error bars. However, Young’s modulus increased significantly, from 5.16 (±0.49) GPa to 9.10 (±0.35) GPa—nearly 1.7 times higher than that of the untreated fibers. According to [27], when CNF dispersions were highly oriented, the resulting materials exhibited both high toughness (~28–31 MJ/m^3^) and high stiffness (~19–20 GPa), along with high yield strength (~130–150 MPa). In contrast, the CNF-treated fibers in this study exhibited a Young’s modulus of 9.10 (±0.35) GPa and did not achieve the ~40% modulus increase reported in highly oriented systems. This discrepancy is likely due to insufficient CNF orientation and the limited thickness of the applied CNF layer, which prevented the material from achieving sufficient strength.

We carried out the washing test according to a standard washing test (JIS L 1930 C4N 140) [19]. The shrinkage ratios are 3.1% and 1.3% for regenerated cellulose fibers and CNF-treated fibers with 5 times washing tests, and 4.1% and 3.0% with 10 times washing tests. The shrink-proof effect remains even after 10 times washing tests, but the effect reduced. The effect is not permanent and is expected to gradually deteriorate over time due to the adhesion of CNF to the fiber surface with physical adsorption effects.

### 3.2. SEM Images of Fiber Surface

**Figure 2:**
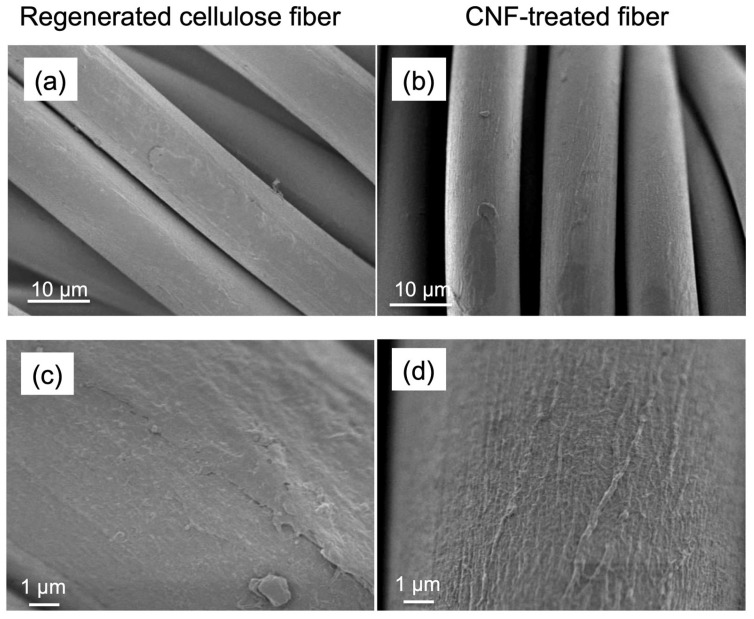
SEM images of (**a**,**c**) regenerated cellulose fibers and (**b**,**d**) CNF-treated fibers under different magnifications: (**a**,**b**) 2000×, and (**c**,**d**) 10,000×.

The fiber surfaces were analyzed using SEM. Figure 2a–d present SEM images of untreated and CNF-treated regenerated cellulose fibers at various magnifications. Figure 2a,b clearly illustrate the general morphology and fibril size distribution, which ranges from 5–20 µm in diameter. Figure 2a shows that the surfaces of the untreated regenerated cellulose fibers appear relatively smooth, whereas Figure 2b reveals thread-like structures on the surfaces of CNF-treated fibers, likely resulting from the adsorption of CNFs. Notably, the overall fiber diameter remains largely unchanged after CNF treatment. Figure 2c,d show higher-magnification SEM images. Figure 2c highlights the presence of cracks and surface roughness of the regenerated cellulose fibers. Conversely, Figure 2d shows thread-like structures adsorbed onto the surfaces of CNF-treated fibers. These structures have diameters ranging from approximately 30 to 150 nm and exhibit a relatively large distribution by Image J 1.54p (National Institute of Health, Bethesda, MD, USA). In addition, they appear to be several microns. These findings support the conclusion that dozens or more CNFs have aggregated on the fiber surface due to the diameter of CNF of 3 nm, and almost completely be covered on the surface of regenerated cellulose fibers following treatment.

### 3.3. Microbeam XRD Measurements

**Figure 3:**
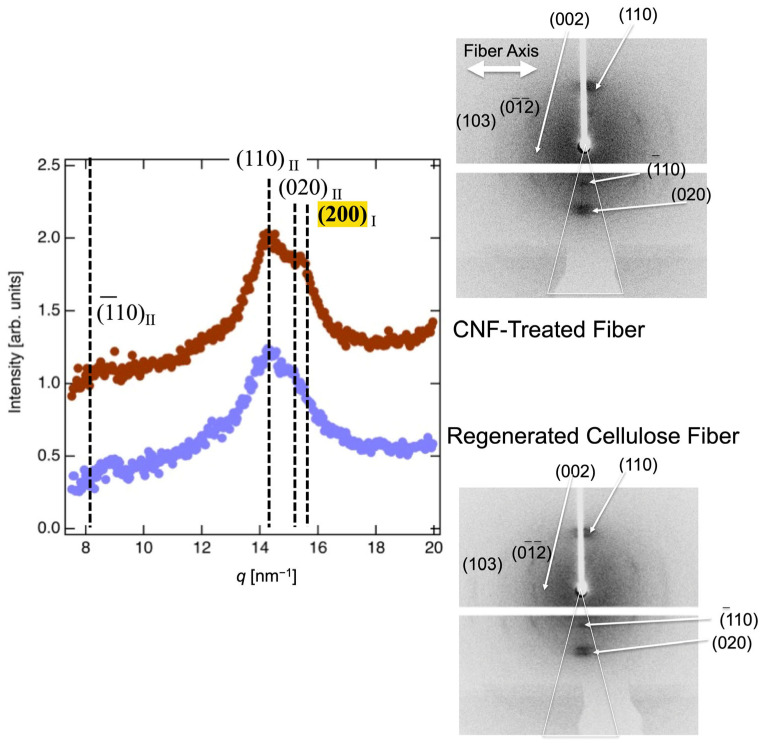
(**left**) XRD profiles of the regenerated cellulose fiber (purple dots) and the CNF-treated fiber (brown dots). The fiber axis is in the lateral direction. The baseline of each profile is shifted up or down to avoid overlap. (**right**) 2D image of XRD profile of regenerated cellulose fiber and CNF-treated fiber. The fiber axis is in the lateral direction.

Microbeam XRD measurements were used to analyze the crystal structures of cellulose in both regenerated and CNF-treated fibers. On the right-hand side of Figure 3, the two-dimensional (2D) images obtained using microbeam XRD for regenerated cellulose fiber and CNF-treated fiber can be seen. These images depict typical fiber diffraction patterns consistent with cellulose II crystals, which are characteristic of regenerated cellulose fibers [28,29,30]. These profiles are caused by highly oriented crystal structure, such as fiber. A broad, weak, amorphous halo is also visible, indicating the presence of non-crystalline regions. For a more detailed analysis, the left-hand side of Figure 3 shows the XRD profiles calculated in the direction normal to the fiber axis, averaged across a 30° sector. The data were corrected for background scattering and transmittance. Distinct diffraction peaks at 8.67, 14.1, and 15.1 nm^−1^ were observed and assigned to the (1-10), (110), and (020) reflections, respectively, which are characteristic of cellulose II crystals present in both regenerated and CNF-treated fibers [31]. Furthermore, the degree of crystallization was calculated to be 60% using the method of Isogai and Usuda [32]. In addition, a weak peak was observed at 15.5 nm^−1^ in the CNF-treated fiber sample and attributed to cellulose I crystals originating from the CNF component [33,34]. Figure S2 shows the FT-Raman spectra of the fiber samples. In both regenerated and CNF-treated fibers, strong peaks associated with cellulose II at 577 cm^−1^ are observed [34]. Conversely, no cellulose I peak is evident in the CNF-treated fibers, suggesting a small presence of the CNF component, which is known to exhibit high crystallinity in the cellulose I form, as observed in the XRD results.

**Figure 4:**
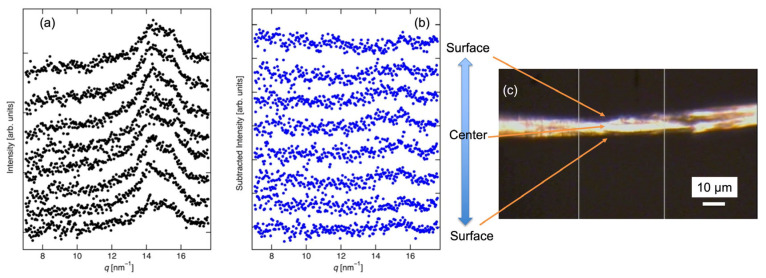
(**a**) XRD profiles at each position of CNF-treated fiber. (**b**) XRD intensity of CNF when the regenerated fiber’s intensity was subtracted, at each position of the CNF-treated fiber. The measurements were conducted at every 1.5 μm. (**c**) Optical micrograph of a fiber sample.

To evaluate the distribution of CNF on the surface of the regenerated cellulose fibers, microbeam XRD profiles were measured by scanning from the surface to the center of the fiber and back. Figure 4a shows the XRD profiles obtained during this scan. Diffraction peaks corresponding to the (200) reflection of cellulose I crystals were observed, along with a peak at 15.5 nm^−1^, in all CNF-treated fiber samples. However, the scattering intensity near the surface was too weak to allow accurate structural evaluation. To enhance the analysis, differential scattering intensity was calculated by subtracting the XRD intensity of the regenerated cellulose fibers from that of the CNF-treated fibers. These results, shown in Figure 4b, reveal that cellulose I crystals were present across all regions of the fiber, although no distinct surface-layer structural deviation due to CNF was detected. From SEM images, the thickness of CNF layers on the surface would be 30–150 nm in Figure 2 and CNF was thinly distributed on the regenerated cellulose fiber surface owing to the CNF treatment. Furthermore, the CNF layer, 30–150 nm, was significantly thinner than the beam size, 500 nm × 500 nm. Figure 4c shows the optical micrograph of a fiber sample.

### 3.4. Higher-Ordered Structure Between Dried and Wet Conditions

**Figure 5:**
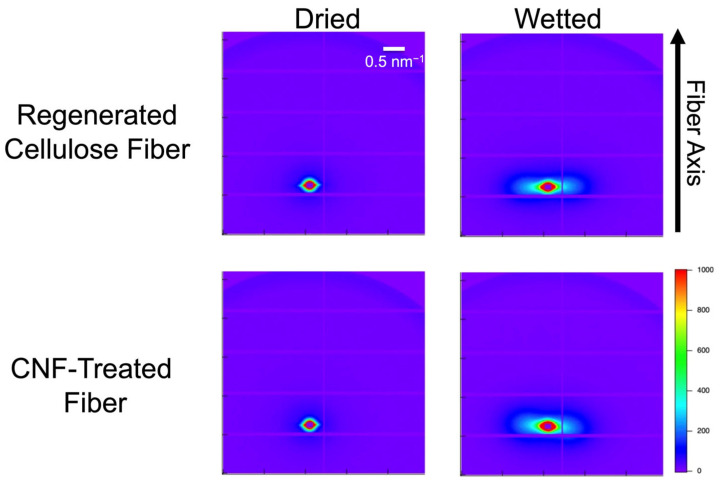
2D SAXS profiles of regenerated cellulose fibers and CNF-treated fibers under dried and wet conditions. The fiber axis is in the meridional direction. Under wet conditions, the strong streak-like scattering profiles were observed normal to the fiber axis.

The presence of CNF on the fiber surface significantly affects the water absorption behavior and humidity dependence of regenerated cellulose fibers. To validate this effect, SAXS measurements were performed to compare the internal structures of regenerated and CNF-treated fibers in both dry and water-absorbed states. Figure 5 shows the corresponding 2D scattering patterns for each sample, with the fiber axis oriented longitudinally. In the dry state, all samples exhibit weak streak-like scattering along the fiber axis. Upon water absorption, the scattering intensity perpendicular to the fiber axis increased significantly.

**Figure 6:**
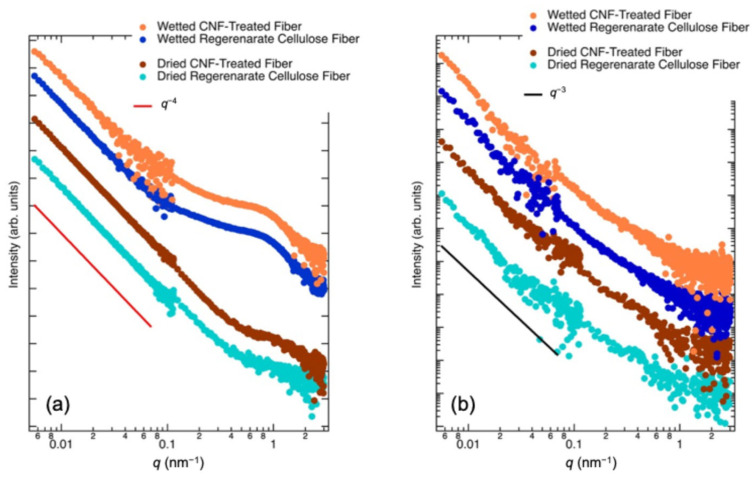
SAXS profiles of regenerated cellulose fibers and CNF-treated fibers under dry and wet conditions: (**a**) normal to the fiber axis and (**b**) parallel to the fiber axis. The solid lines in (**a**,**b**) represent slopes of *q*^−4^ and *q*^−3^, respectively.

This type of streak-like scattering resembles the profiles reported by [28,34] for regenerated cellulose fibers such as viscose and lyocell. However, Sharma et al. observed that the microvoid distribution parallel to the fiber axis was minimal [35]. This is likely due to the absence of crystal-derived scattering associated with lamellar structures aligned along the fiber axis. To analyze this further, sector averages were calculated for each fiber type. The results are presented in Figure 6.

Regarding the scattering profiles perpendicular to the fiber axis, both regenerated cellulose fibers and CNF-treated fibers, whether dry or water-absorbed, show a decreasing trend proportional to *q*^−3^. Starting at approximately *q* = 0.4 nm^−1^, this trend transitions to a *q*^−2^ dependence. This indicates minimal scattering from lamellar structures oriented along the fiber axis. Consequently, these fibers are inferred to consist of very long fibrillar structures with largely absent lamellar structures of approximately 10 nm. Focusing on the scattering parallel to the fiber axis, the scattering curves for all fiber types decrease to ~*I*(q)–*q*^−4^ at *q* below 0.4 nm^−1^. This behavior is attributed to scattering from microvoid interfaces [27,36]. In contrast, in this study, no scattering from lamellar structures was observed, and the regenerated cellulose fibers displayed a fringed fibrillar structure parallel to the fiber axis. As shown in Figure 2, SEM images reveal no lamellar-type structures. Furthermore, upon water absorption, scattering peaks appeared at approximately *q* = 0.4 nm^−1^ corresponding to microvoid formation. The increased scattering intensity in the presence of water suggests that the diameter of the microvoids expands as water molecules are absorbed.

**Figure 7:**
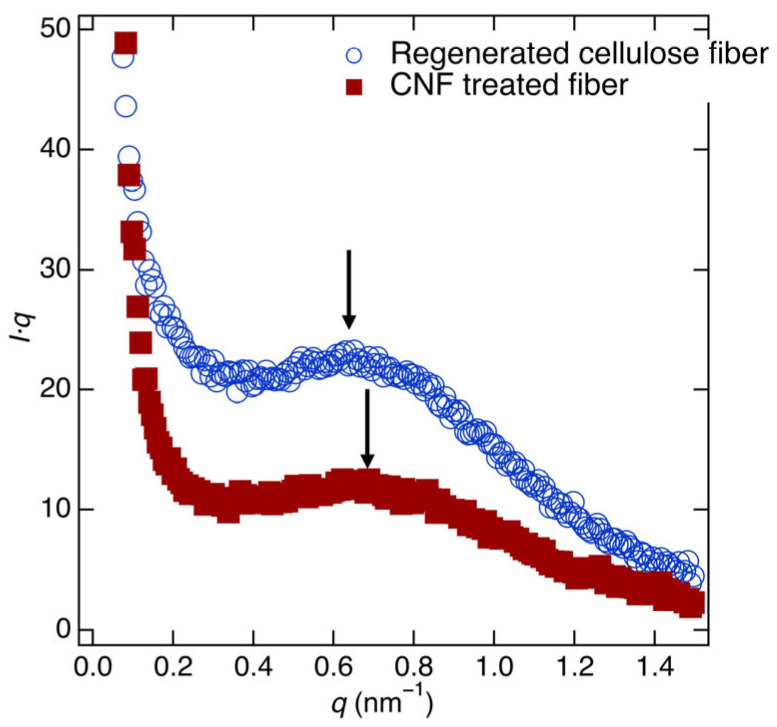
Lorentz-corrected SAXS profiles of wet regenerated cellulose fibers (blue open circles) and wet CNF-treated cellulose fibers (red full squares). The arrows show the peak position of these profiles. The arrows show the peak position of both fiber samples.

To evaluate the correlation length of the fibril-type structures, *I*(*q*) was multiplied by *q*, following the Lorentz correlation method [37,38]. Figure 7 shows the Lorentz-corrected scattering profiles of wetted regenerated cellulose fibers and wetted CNF-treated fibers. The peak positions were observed at *q* = 0.621 (±0.007) nm^−1^ for the regenerated cellulose fibers and *q* = 0.655 (±0.008) nm^−1^ for the CNF-treated fibers. These correspond to correlation lengths of 10.11 (±0.11) nm and 9.58 (±0.12) nm, respectively. These findings indicate that the CNF treatment results in a smaller correlation length, which is consistent with the observed reduction in swelling upon water absorption.

### 3.5. FT-IR Spectra of Various Humidity Conditions

**Figure 8:**
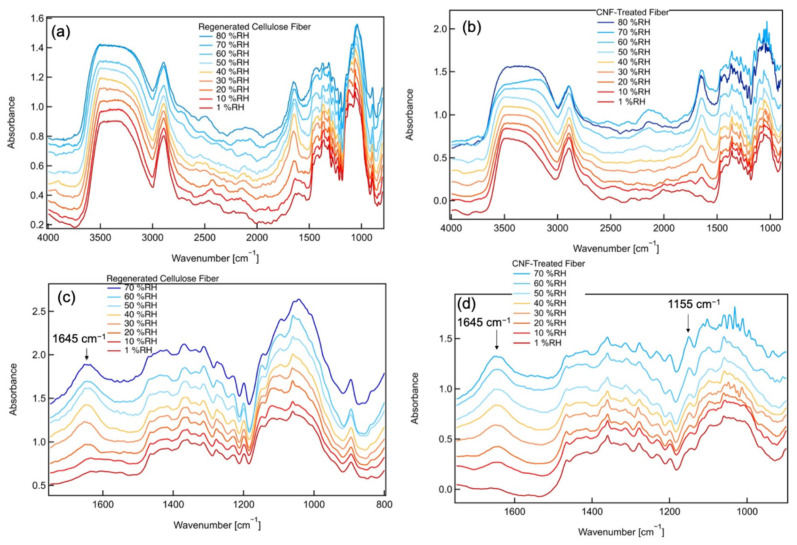
Humidity dependence of the FT-IR spectra between 4000 and 800 cm^−1^ in (**a**) regenerated cellulose fiber and (**b**) CNF-treated fiber. Expanded FT-IR spectra between 1800 and 900 cm^−1^ in (**c**) regenerated cellulose fiber and (**d**) CNF-treated fiber. The arrows correspond to 1645 cm^−1^ for the OH stretch (ν(OH)) of cellulose/water molecules and 1155 cm^−1^ for the C-O-C asymmetric stretching vibration at the β-glucoside linkage in the wetted CNF film [41,43].

Microbeam FT-IR measurements revealed the humidity dependence of the FT-IR spectra of each fiber, as shown in Figure 8a,b. The peak at approximately 3400 cm^−1^ corresponds to the OH stretching vibration (ν(OH)) of both cellulose and water molecules [39]. It is difficult to distinguish between the contributions of cellulose I, cellulose II, and water is difficult due to overlapping absorbance bands. An absorption band appears at 2892 cm^−1^, assigned to the vibrations of CH_2_ (ν(CH)). This band remained unchanged across different humidity levels [25]. These results suggest that the CH_2_ groups in the main chain are significantly affected by hydrogen bonding.

**Figure 9:**
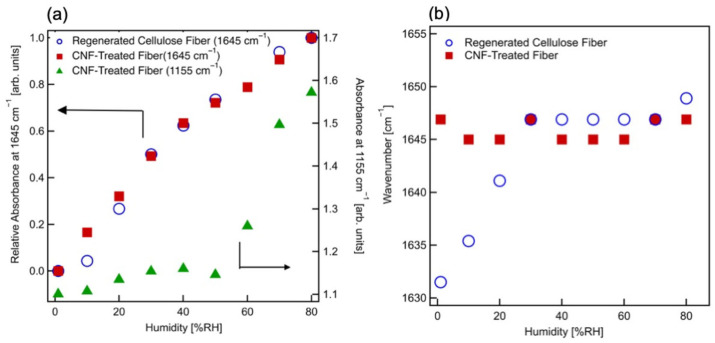
(**a**) Humidity dependence of relative absorbance at 1645 cm^−1^ for the regenerated cellulose fiber (blue open circle), and the CNF-treated fiber (red filled square), and absorbance at 1155 cm^−1^ for the CNF-treated fiber (green filled triangle). (**b**) Humidity dependence of wavenumber around 1645 cm^−1^ for vibration bands of H2O molecules, δ(HOH), for the regenerated cellulose fiber (blue open circle), and the CNF-treated fiber (red filled square).

For a more detailed analysis, Figure 8c,d show the expanded FT-IR spectra of the regenerated cellulose fiber and the CNF-treated fibers in the 1750–800 cm^−1^ range. Some reports have suggested characteristic bands of cellulose I (natural cellulose) at 1359 and 1103 cm^−1^ [40,41], but it is difficult to discuss these bands due to overlapping between the water and cellulose II bands. Therefore, we focused on the peak intensity at 1645 cm^−1^, corresponding to the vibration of water molecules (δ(HOH)) [42], which increases gradually with increasing humidity. These results suggest that cellulose molecules absorb water molecules through strong hydrogen bonding interactions. Figure 9a,b present the humidity dependence of both the absorbance intensity and the peak position at 1645 cm^−1^. For a detailed analysis, the spectra at various humidity conditions were subtracted from the intensity at 1% RH and normalized to the intensity at 80% RH. Therefore, the peak absorbance increased almost linearly with humidity, and the humidity dependence of the relative absorbance was nearly identical for both fiber types. These results suggest that water uptake influences the molecular structure of the regenerated cellulose fiber components.

Conversely, the wavenumber shift corresponding to the peak absorbance at 1645 cm^−1^ differed between regenerated cellulose fibers and CNF-treated fibers, as shown in Figure 8b. For CNF-treated fibers, the wavenumber remained independent of humidity, whereas for regenerated cellulose fibers, it increased with humidity up to 30% RH. This behavior below 30% RH reflects the humidity-dependent hydrogen bonding of water molecules. Figure 8d shows that the absorbance intensity at 1155 cm^−1^ increases with humidity [41,43]. However, no absorbance peak at this wavenumber was observed in the regenerated cellulose fiber. This band is attributed to the C-O-C asymmetric stretching vibration of the β-glucoside linkage [44], which is characteristic of the cellulose chain. The effect of humidity on this band in CNF films has also been reported [41]. Accordingly, we evaluated the humidity dependence of the peak intensity at 1155 cm^−1^ of the CNF-treated fiber, as shown in Figure 9b. The peak intensity increased gradually at RH levels up to 50%, then decreased sharply above 60% RH. This suggests that under high humidity, CNF on the fiber surface absorbs water molecules and swells. We speculated that this expansion inhibits further water absorption by the regenerated cellulose fibers, thereby reducing swelling and increasing the elastic modulus during water absorption. Due to the diffraction of infrared light, the beam size in microbeam FT-IR measurements is 5 μm square, which makes it impossible to analyze smaller regions. In addition, due to the small size of CNF, it is currently not feasible to directly evaluate the interactions between CNF and regenerated cellulose in CNF-treated fibers. Further analysis and a deeper investigation are required for a more detailed understanding.

### 3.6. Schematic Drawing of Water Absorption

**Figure 10:**
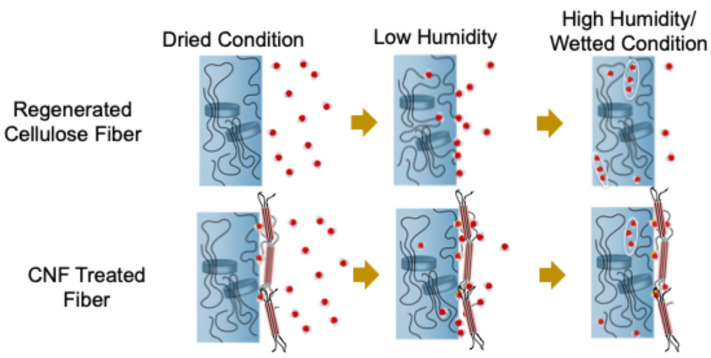
Schematic drawing of regenerated cellulose fiber and CNF-treated fiber in the case of dried, low-humidity, and high-humidity/wetted conditions. Blue and red fibers are regenerated fibers and CNF on the surface of regenerated fibers.

In summary, CNF treatment significantly affected the surface characteristics of the regenerated cellulose fibers. Figure 10 illustrates the water adsorption behavior and the impact of CNF treatment. At relatively low humidity, water molecules were primarily absorbed into the fibrils of the regenerated cellulose fiber, regardless of the CNF treatment. Under high-humidity or wet conditions, water clusters formed within the regenerated cellulose fibers. In contrast, for CNF-treated fibers, the number of water molecules adsorbed on the CNF surface increased sharply with humidity. The CNF-treated fiber has a high crystallinity surface (70%); therefore, the CNF layer on the surface prevents water from being absorbed into the regenerated cellulose fibers (crystallinity ~60%). Furthermore, the humidity-dependent behavior inhibits water absorption into the regenerated cellulose fibers when immersed in water. This reduces swelling and suppresses the degradation of the physical properties of the fibers associated with water absorption.

In this study, after five washing machine tests, regenerated cellulose fiber shrinks by 3.1%, while CNF processing reduces this to 1.3%. Even after 10 washing machine tests, regenerated cellulose fiber shrinks by 4.1%, whereas CNF processing results in a shrinkage of 3.0%. It is necessary to improve durability during washing in a washing machine; therefore, further structural analyses attribute these improvements to the presence of highly crystallized CNF adsorbed on the fiber surface. Our research shows that there is a strong interaction between fibers and CNF, which greatly affects water adsorption and absorption. However, the nature of this interaction could not be fully elucidated in this study. Since water is not absorbed into CNF unless the relative humidity exceeds 60%, water could be easily adsorbed onto the CNF surface. To clarify this interaction between the regenerated cellulose fiber and CNF, further experiments using other cellulose materials and synthetic fibers are necessary.

## 4. Conclusions

The results obtained in this study clarify the effects of CNF treatment on the mechanical performance and water resistance of regenerated cellulose fibers. We elucidated the impact of CNF treatment on the mechanical properties and water resistance of regenerated cellulose fibers. The incorporation of CNF onto the fiber surface resulted in an enhancement of Young’s modulus by approximately 1.5 times compared to untreated regenerated cellulose fibers, although this value remains at about 30% of the modulus observed in CNF alone. Furthermore, the presence of CNF led to a reduction in water absorption of regenerated cellulose fibers. To investigate the structural modifications induced by CNF processing, SEM images confirmed the existence of CNF on the regenerated cellulose fiber surface, while XRD measurements revealed that CNF is oriented along the axis of the regenerated cellulose fiber. Structural changes upon water uptake were examined using X-ray scattering and FT-IR spectroscopy. X-ray scattering indicated that CNF processing diminished the thickness of nanometer-scale water layers absorbed within the regenerated cellulose matrix. Conversely, FT-IR results showed that, under low-humidity conditions, the water-associated band near 1645 cm^−1^ exhibited a shift to higher wavenumbers, suggesting the occurrence of specific interactions between CNF and water molecules at low humidity. Additionally, it was determined that substantial water adsorption onto CNF occurs at relative humidities of 60% or higher. Collectively, these results indicate that surface-localized CNF inhibits the absorption of water molecules into the regenerated cellulose fiber matrix. Notably, the presence of CNF at the fiber surface not only enhanced mechanical elasticity but also significantly influenced water adsorption behavior, thereby suppressing fiber swelling.

This research establishes that the application of CNF processing to regenerated cellulose fiber materials—otherwise vulnerable to water and humidity—significantly enhances their water and moisture resistance. Detailed structural analyses attribute these improvements to the presence of highly crystallized CNF adsorbed on the fiber surface.

Consequently, this advancement suggests the feasibility of laundering regenerated cellulose fibers, derived from natural sources, in conventional washing machines. Such a development could substantially broaden the commercial applicability of these materials.

## Data Availability

Raw data were generated at Yamagata University. Derived data supporting the findings of this study are available from the corresponding author, G.M., upon request.

## Supplementary Materials

The following supporting information can be downloaded at: https://www.mdpi.com/article/10.3390/polym17152015/s1, Figure S1: Clothing made from each fiber samples before and after washing by washing machine in water. Figure S2: FT-Raman spectra of each fiber sample.

## Abbreviations

The following abbreviations are used in this manuscript:

CNF	Cellulose nano fiber
SEM	Scanning electron microscopy
SAXS	Small-angle X-ray scattering
FT-IR	Fourier transform infrared
XRD	X-ray diffraction
RH	Relative humidity
