# Supplementary material for: Structural Analysis of Regenerated Cellulose Textile Covered with Cellulose Nano Fibers
Source: Polymers (Basel). 2025 Jul 23;17(15):2015. doi: 10.3390/polym17152015 (PMC12349284; doi:10.3390/polym17152015)
Supplement: Supplementary file 1 [file polymers-17-02015-s001.zip › polymers-3751135-supplementary.pdf]

Supporting Information

Structural analysis of regenerated cellulose textile covered with cellulose nano fibers

Ayaka Yamaji[1], Yui Okuda[1], Chikaho Kobayashi[1], Rikako Kurahashi[1], Kyoko Kazuma[2], Kazuki Chiba[2], Mitsuhiro Hirata[2], Yuka Ikemoto[3], Keiichi Osaka[3], Jiacheng Gao[4], Harumi Sato[4], and Go Matsuba[1,*]

[1] Graduate School of Organic Material Science, Yamagata University, 4-3-16 Jonan, Yonezawa Yamagata 992-8510, Japan.
[2] Yamagata Research Institute of Technology, 2-2-21 Shoei, Yamagata-city, Yamagata 990-2473, Japan.
[3] JASRI/SPring-8, 1-1-1 Koto, Sayo-cho, Hyogo 679-5198, Japan.
[4] Graduate School of Human Development and Environment, Kobe University, 3-11 Tsurukabuto, Nada-ku, Kobe, Hyogo 657-8501, Japan.
*Correspondence: gmatsuba@yz.yamagata-u.ac.jp

Characteristic properties of CNF treatments for regenerated cellulose fiber

Figure S1 shows the changes in clothing made from regenerated cellulose fibers and CNF-treated fibers before and after washing with water. Cloths made from regenerated cellulose fibers shrinks after washing, whereas cloths made from CNF-treated fibers shows no shrinkage.

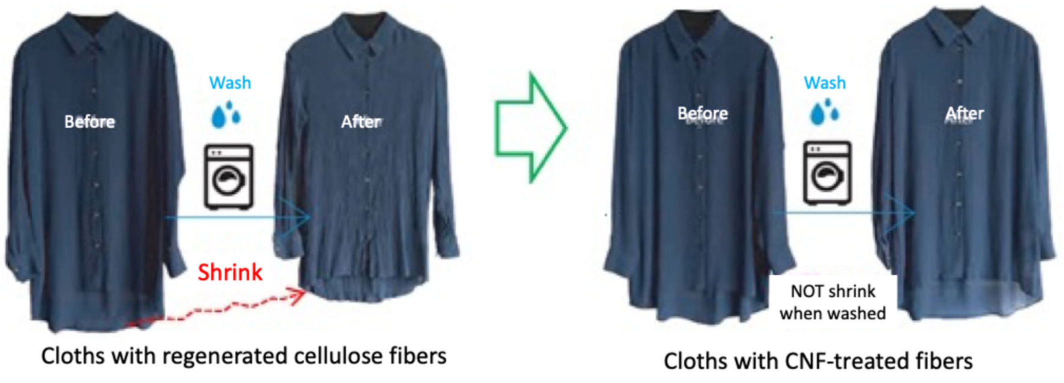

Figure S1. Cloths with regenerated fibers and CNF-treated fibers before / after washing in washing machine with water.

FT-Raman spectra

Figure S2 shows the FT-Raman spectra of regenerated cellulose fibers and CNF-treated fibers. Dassanayake et al. assigned some peaks of FT-Raman spectra [20]. The peak at 577 cm^{-1} assigned as cellulose II crystal is observed in both fibers. However, it is difficult to observe any peaks from cellulose I crystals of 1120 cm^{-1} and 1476 cm^{-1}. These results suggest that both fibers compose almost as regenerated cellulose component and CNF component is very little in CNF-treated fibers.

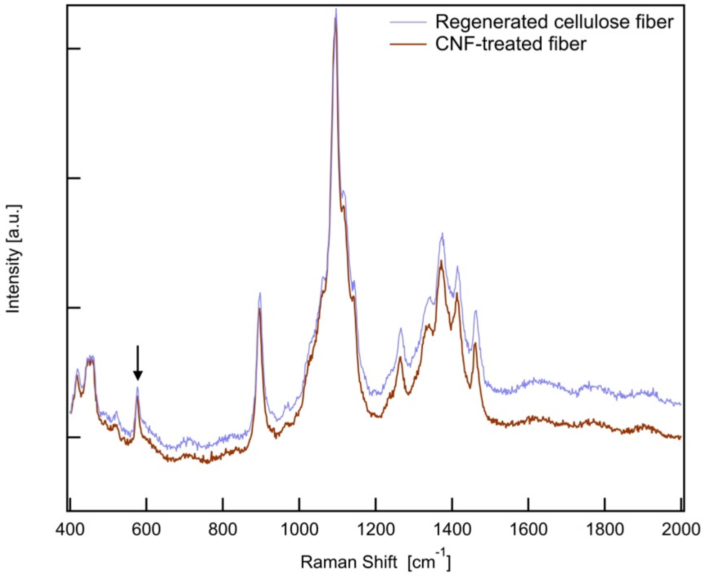

Figure S2. FT-Raman spectra of regenerated cellulose fiber and CNF-treated fiber.
